# Microcirculation After Trochanteric Femur Fractures: A Prospective Cohort Study Using Non-invasive Laser-Doppler Spectrophotometry

**DOI:** 10.3389/fphys.2019.00236

**Published:** 2019-03-25

**Authors:** Bergita Ganse, Franziska Böhle, Tatjana Pastor, Boyko Gueorguiev, Simon Altgassen, Gertraud Gradl, Bong-Sung Kim, Ali Modabber, Sven Nebelung, Frank Hildebrand, Matthias Knobe

**Affiliations:** ^1^Department of Orthopaedic Trauma Surgery, RWTH Aachen University Hospital, Aachen, Germany; ^2^Department of Orthopaedics, Balgrist University Hospital, University of Zürich, Zurich, Switzerland; ^3^AO Research Institute Davos, Davos, Switzerland; ^4^LVR Hospital Essen, University Duisburg-Essen, Essen, Germany; ^5^Department of Plastic and Reconstructive Surgery, Hand Surgery - Burn Center, RWTH Aachen University Hospital, Aachen, Germany; ^6^Department of Oral and Maxillofacial Surgery, RWTH Aachen University Hospital, Aachen, Germany; ^7^Department of Radiology, RWTH Aachen University Hospital, Aachen, Germany

**Keywords:** microcirculation, fracture, trauma, SO_2_, smoking, nicotine, age, diabetes

## Abstract

Proximal femur fractures represent a major healthcare problem in the aging society. High rates of post-operative infections are linked to risk factors that seem to affect local microcirculation. Patterns and time courses of alterations in microcirculation have, however, not been previously investigated. The aim of this prospective cohort study was to evaluate perioperative changes in microcirculation after trochanteric femur fractures using non-invasive laser-Doppler spectrophotometry to analyze how oxygen saturation (SO_2_), hemoglobin content (Hb) and blood flow changed before and after surgery, and how these parameters were altered by implant type, gender, smoking, diabetes and age. Measurements were separately recorded for nine locations around the greater trochanter in 2, 8, and 15 mm depths, before surgery and 8, 24, 48 h, 4, 7, and 12 days after surgery in 48 patients. Three implants were compared: Dynamic Hip Screw, Gamma3 Nail, and Percutaneous Compression Plate. Surgery resulted in significant differences between the healthy and injured leg in SO_2_, Hb and flow. Each parameter showed comparable values for both legs prior to surgery. Significantly higher values in SO_2_ and flow were registered in women compared to men before and after surgery. Smoking caused significant increases in SO_2_, Hb, and flow only in the superficial layer of the skin after surgery. Diabetes decreased blood flow at 2 and 8 mm depth and increased SO_2_ at 8 and 15 mm depth after surgery. Age revealed a significant negative correlation with flow. The ability to increase the flow rate after surgery decreased with age. Comparison of implants indicated the minimally invasive implant PCCP altered microcirculation less than the DHS or the Gamma3 nail. Overall, the proximal femur fracture alone did not alter local skin microcirculation significantly in a way comparable to the effect caused by surgery. In conclusion, microcirculation after proximal femur fractures is highly affected by surgery, gender, smoking, diabetes, age and implant in ways specified in this study.

## Introduction

Fractures of the proximal femur are associated with high morbidity and mortality and represent a major healthcare problem in the aging society (Knobe and Siebert, 2014; Neuerburg et al., 2015; Carow et al., 2017). Post-operative rates of surgical site infection after surgery of proximal femur fractures were reported between 1.7 and 10% in arthroplasty or hemiarthroplasty and under 3% in femur nails, dynamic hip screws and minimally invasive implants (Knobe et al., 2009, 2012, 2013; Noailles et al., 2016; de Jong et al., 2017). Known risk factors for wound infection include a body mass index higher than 35, male gender, diabetes mellitus, multiple previous incisions, lymphoedema, poor vascular perfusion, inflammatory arthropathy, renal or liver disease, immune compromise, corticosteroid therapy, smoking, and poor nutrition (Jones et al., 2013; Namba et al., 2013; Kong et al., 2017). Microcirculation is usually altered after trauma (Ljung et al., 1995; Soneja et al., 2005). Most risk factors are associated with inhibition in local microcirculation and subsequent decrease in oxygen supply, hypoxia, acidosis, collection of metabolites and oxidative/nitroxidative stress (Soneja et al., 2005; Bentov and Reed, 2014). Local capillarization is not primarily dictated by local oxidative capacity, but rather by factors such as substrate delivery and metabolite removal (Bosutti et al., 2015). Impairment of the local function of the immune system supports bacterial growth (Bentov and Reed, 2014). Connections between microcirculation and risk of infection were established on a molecular level in recent years (Eming et al., 2017), however, clinical evidence in an orthopedic surgery context is still rare. Especially the link between rates of infection and decreased microcirculation is still missing. Ljung et al. (1995) used laser-Doppler imaging to detect increased skin microcirculation in five patients after elbow prosthesis, concluding no impairment after elbow arthroplasty. In previous studies, differences in microcirculation were found between surgical approaches in spine surgery, i.e., less impairment of microcirculation in minimally invasive surgery of the spine compared to open surgery (Ganse et al., 2017). Distinctions in microcirculation between approaches in Achilles tendon surgery in healthy subjects (Klos et al., 2018), gender differences in skin microcirculation over the calcaneus (Carow et al., 2018) and effects of pulsed ultrasound therapy in the foot were published (Kösters et al., 2017).

Several technologies and devices are available to measure factors determining microcirculation (Eriksson et al., 2014). The use of laser-Doppler spectrophotometry was first described in 1998 and is clinically applied in flap surgery, but also for assessment of diabetic ulcers or burning injuries (Forst et al., 2008; Merz et al., 2010; Rothenberger et al., 2013; Kneser et al., 2014; Mucke et al., 2014). Since then, it has been widely used in research for investigations of different types of tissues (Forst et al., 2008; Fechner et al., 2009; Klein et al., 2011).

At this stage, we see a high demand in further studies to understand which parameters and characteristics of microcirculation increase the risk for infection after orthopedic surgery. Knowledge on differences in microcirculation between implants would be important for the choice of surgical implants and the development of new implant types. Especially the question how microcirculation differs between conventional and minimally invasive implants is of high clinical importance, particularly in the context of geriatric patients.

Patterns and time courses of changes in microcirculation after proximal femur fractures and their alterations through risk factors have not been previously investigated. Moreover, differences in microcirculation after fixation with different implant types have not been reported either. Implants include intramedullary nails (example: Gamma3 Nail, Proximal Femoral Nail Antirotation), plates (example: Dynamic Hip Screw, DHS), minimally invasive implants (example: Percutaneous Compression Plate, PCCP) or arthroplasty/hemiarthroplasty. Therefore, we conducted the present study with the aim to fill in this gap of knowledge and test the hypothesis that diabetes, smoking, age, gender, implant type and proximal femur fracture do not affect cutaneous microcirculation of the tissue surrounding the proximal femur.

## Materials and Methods

This study was carried out in accordance with the recommendations of the Committee on Publication Ethics (COPE) and the International Committee of Medical Journal Editors (ICMJE). The protocol was approved by the RWTH Aachen University Hospital IRB (reference number EK006/11, date of approval: 15.04.2011). All subjects gave written informed consent in accordance with the Declaration of Helsinki. The prospective cohort study was registered in www.ClinicalTrials.gov (number NCT01264172) with the title “Identification of Microcirculation and Inflammation After Minimally-invasive Osteosynthesis of the Proximal Femur” (MicroProxFem).

### Study Design

Inclusion criterion was presentation to the emergency room of RWTH Aachen University Hospital with traumatic trochanteric femur fracture. Exclusion criteria were pathological fractures, metabolic bone diseases, prior hip or femur surgeries, leg deformities, soft tissue damage, surgical intervention later than 3 days after the accident, immunodeficiency, polytrauma and fractures extending more than 5cm distal to the lesser trochanter. Patients were not randomized regarding the used surgical implant, however, the operating surgeons selected one out of three implants according to the fracture morphology: dynamic hip screw (DHS, DePuy Synthes, West Chester, Pennsylvania, United States), intramedullary nail (Gamma3, Stryker Corporation, Kalamazoo, United States) or Percutaneous Compression Plate (PCCP, Orthofix, Lewisville, TX, United States). The surgeons were not informed about the patients’ inclusion in the study, which means that their decisions on implant selection were independent from the study.

### Measurements

**FIGURE 1:**
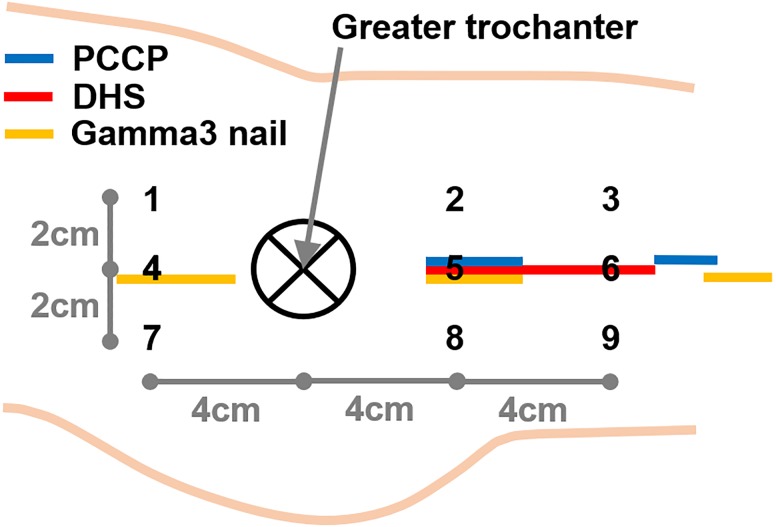
Locations of the measurement points (numbers) and incisions (colored marks) in relation to the greater trochanter.

To assess cutaneous microcirculation, a laser-Doppler spectrophotometry system (“Oxygen to see,” O_2_C, LEA Medizintechnik, Winchesterstr. 2, D-35394 Gießen, Germany) was used with non-invasive fixation of a LH-2 fiberglass probe on the patients’ skin and incorporating a laser (wavelength 820 nm) and a detector in the white-light spectrum range (wavelength 500–800 nm) to measure the following three parameters characterizing microcirculation (Forst et al., 2008): (1) Oxygen saturation (SO_2_) in small capillaries (diameters less than 0.05 mm) via the white-light spectral shift. (2) Local hemoglobin amount (Hb) accounting for the filling of post-capillary venules – via the white-light absorption. (3) Erythrocyte speed and blood flow (flow) in the capillary system (volumetric flow rate) – via the white-light spectral shift. Measurement values were separately recorded for 2, 8, and 15 mm depths. As the volume where each measurement was performed depends on tissue density and device wavelength, it cannot be reported precisely. Baseline data was collected in the operation theater right before surgery. Six measurements following the surgery were performed after 8, 24, 48 h, 4, 7, and 12 days. The measurements obtained from the healthy and injured leg before surgery aimed at possible microcirculatory differences induced by the injury. The probe was affixed on the patients’ skin using tape to standardize contact pressure. For all measurements, patients were lying in supine position on their back. At the injured leg, measurements were taken in 9 locations surrounding the greater trochanter as depicted in Figure 1. Measurement points 1, 4, and 7 were on and next to the proximal incision of the Gamma3 nail, while not being close to any incision of the DHS and PCCP. Measurement points 2, 5, and 8 were on and next to the incisions of the DHS and PCCP, while there was no incision of the nail close. Finally, incisions 3, 6, and 9 were on or close to the distal incision of the DHS. In addition, measurements were performed on the healthy leg using three measurement points (numbers 4, 5, and 6 according to Figure 1). All measurement points were marked on the skin post-operatively to guarantee identical locations. Marking of the points was not possible before surgery, as skin disinfection during surgery would have removed them.

### Statistical Analysis

Statistical analysis was performed using SPSS software package (IBM SPSS Statistics release 20.0.0, Armonk, NY, United States). Flow values bigger than 1000 arbitrary units (AU) were excluded from evaluation. Normal distribution of the collected data was screened and proved with Shapiro–Wilk Test. Multifactorial analysis of variance (ANOVA) was applied with Bonferroni *Post Hoc* Tests for multiple comparisons to analyze influence of gender, smoking, diabetes, time point, measurement point (on skin), depth, implant and leg (healthy vs. injured) on SO_2_, Hb, and flow separately for baseline and post-operative data. One-way ANOVA was applied to explore age of the patients treated with the different implants. Age-related changes in SO_2_, Hb, and flow were analyzed using regression statistics and age as a covariate in ANOVA. Chi-square test was used to investigate the distribution of patients regarding the categories gender (male/female), smoking (yes/no), diabetes (yes/no), and implant type. All data are presented in terms of mean and standard error of mean (SEM). The level of significance was set at 0.05 for all statistical tests.

## Results

Forty-eight patients with a trochanteric femur fracture were successfully recruited in the study between November 2010 and March 2012. Characteristics of the patient collective are given in Table 1. None of the patients developed a post-operative wound infection during the study, which means, here we present the normal course of treatment. All patients were without catecholamines during measurements. Sixteen patients completed all measurements at the 7 time points; 13, 10, and 4 patients missed the last, the last two and the last 3 measurements, respectively. Ten patients did not have a pre-surgery measurement. The reasons for incomplete follow-up measurements were that measurements were obtained during in-patient treatment within our hospital, and patients could not be followed-up when discharged to a different facility. Pre-surgery measurements could only be collected during day-time.

**Table 1:** Characteristics of the recruited patient collective together with *p*-values addressing either the distribution of patients across the respective categories or comparing age of the patients treated with the three different implants.

Variable	DHS (*n* = 12)	PCCP (*n* = 13)	Gamma3 nail (*n* = 25)	Total/average	*p*-value
Male	6	5	12	23	0.869
Female	6	8	13	27	
Smoker	1	4	3	8	0.347
Non-smoker	11	9	22	42	
Diabetes	3	3	3	9	0.449
No diabetes	9	10	22	41	
Average age (years)	71.0 ± 17.8	76.5 ± 14.9	77.5 ± 12.5	75.48 ± 14.4	0.727

*Average age is followed by SD. DHS, dynamic hip screw; PCCP, Percutaneous Compression Plate.*

### No Differences Between Legs Before Surgery

**FIGURE 2:**
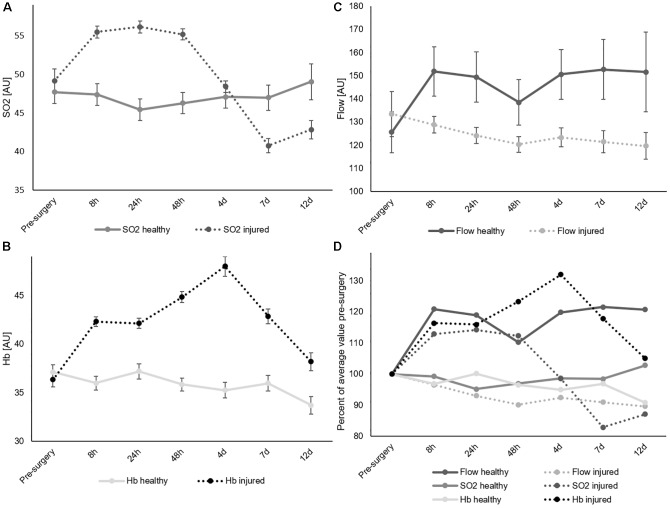
Comparison of healthy and injured leg for SO_2_ **(A)**, Hb **(B)**, and flow **(C)**. All measurement points pooled. Average values. Error bars show SEM. **(D)** Percent changes normalized to the pre-surgery measurement and separated by leg.

**FIGURE 3:**
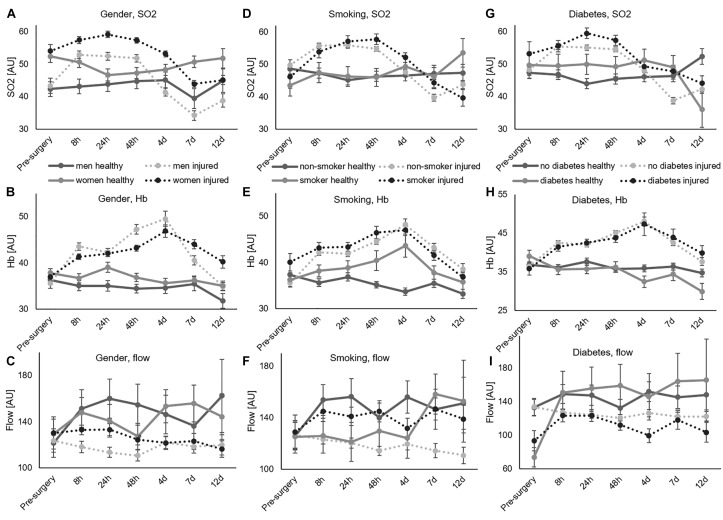
Comparison of healthy and injured leg, error bars show SEM. **(A–C)** Effects of gender displayed for SO_2_, Hb, and flow. **(D–F)** Effects of non-smoking vs. smoking, displayed for SO_2_, Hb, and flow. **(G–I)** Effects of no diabetes vs. diabetes, displayed for SO_2_, Hb, and flow.

Table 2 shows results of the multifactorial ANOVA, indicating no significant pre-operative difference between the healthy and injured leg. Data is displayed in Figure 2. In addition, there were no significant differences between legs in the separate groups of male and female patients, smokers and non-smokers, diabetics and non-diabetics before surgery (Figure 3).

**Table 2:** *P*-values of comparisons among baseline-data (pre-surgery measurements, pre-OP) and post-surgery measurements (post-OP).

	SO_2_ pre-OP	SO_2_ post-OP	Hb pre-OP	Hb post-OP	Flow pre-OP	Flow post-OP
Time points		**<0.001**		0.149		0.276
Implant		0.175		**<0.001**		0.372
Depth	**<0.001**	**<0.001**	**<0.001**	**<0.001**	**<0.001**	**<0.001**
Measurement point	0.366	0.949	0.278	**<0.001**	0.453	**0.001**
Leg	0.305	**<0.001**	0.277	**<0.001**	0.354	**<0.001**
Age	0.755	0.615	**<0.001**	**<0.001**	0.525	**0.001**
Gender	**<0.001**	**<0.001**	**<0.001**	**0.006**	0.534	**<0.001**
Smoking	0.121	0.163	**0.016**	**0.015**	0.266	0.718
Diabetes	0.218	**<0.001**	**<0.001**	0.126	**0.007**	**0.004**

*Gray color highlights significances detected only after surgery (and not before). Significant p-values are shown bolted. Time points after surgery were pooled (8, 24 h, 4, 7, and 12 days after surgery). Implant, comparison of DHS; PCCP, and Gamma3 nail (no implant pre-surgery); depth: 2, 8, and 15 mm; measurement point, the nine measurement points on the skin (Figure 1); leg, healthy vs. injured; smoking, yes/no; diabetes, yes/no. Chi-square test was used for gender, smoking, diabetes, and implant. The other p-values are from multiple regression ANOVA.*

### Effects of Surgery

Table 2 displays *p*-values of comparisons among baseline-data (pre-surgery measurements, pre-OP) and post-surgery measurements (post-OP). Figure 3 depicts the data with average value and SEM comparing the healthy and injured leg for gender, smoking and diabetes over time. Supplementary Figures 6–8 shows the same for each depth level.

**FIGURE 4:**
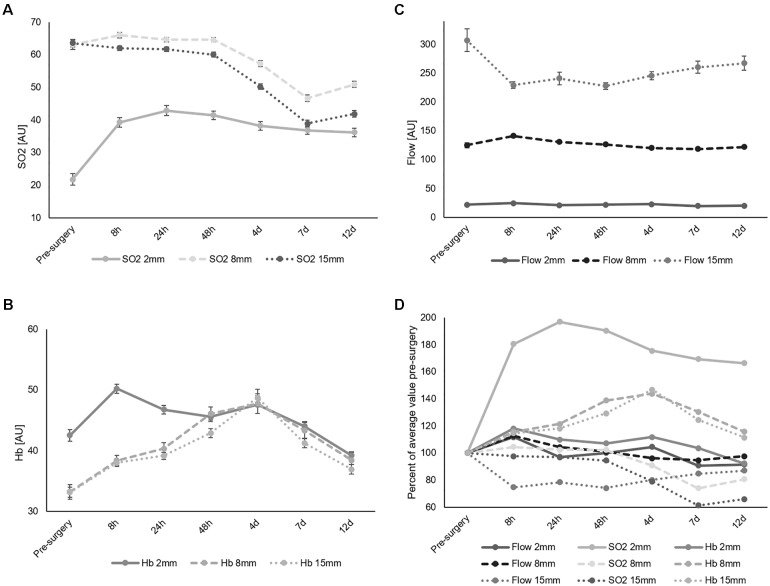
Average measured values of SO_2_ **(A)**, Hb **(B)**, and flow **(C)** of the injured leg separated by depth and time point. The measurements at each time point are pooled. Error bars show SEM. **(D)** Average measured values of SO_2_, Hb, and flow of the injured leg normalized to the pre-surgery measurement and separated by depth and time point.

**FIGURE 5:**
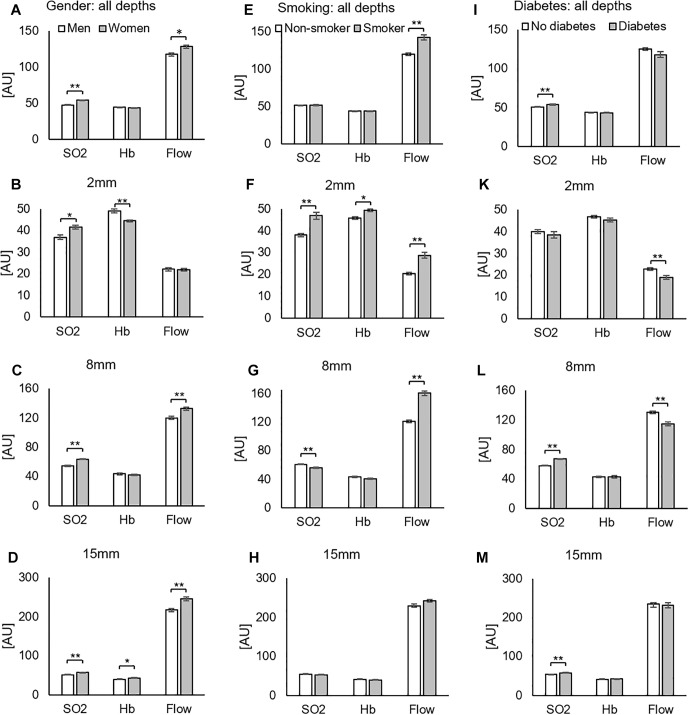
Injured leg, all postoperative measurements. Influence of gender **(A–D)**, smoking **(E–H),** and diabetes **(I–M)** on SO_2_, Hb, and flow for all measured depths together **(A,E,I)** and separated by measurement depth (2 mm: **B,F,K**. 8 mm: **C,G,L**. 15 mm: **D,H,M**). ^∗^*p* < 0.05; ^∗∗^*p* < 0.001 (one-way ANOVA).

With regards to SO_2_, significant differences between different depths (Figure 4A) and gender (Figure 3A) were already present before surgery. The surgery initiated significant SO_2_ differences between healthy and injured legs (Figure 2A, 3) as well as between diabetic and non-diabetic patients (Figure 3G, 5I–M).

**FIGURE 6:**
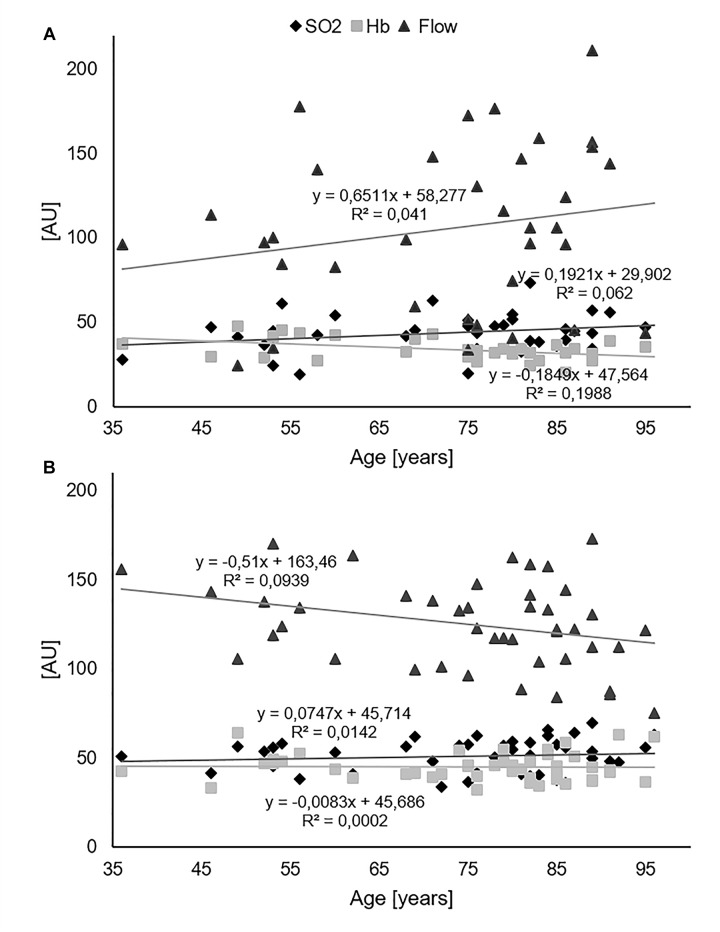
Regression analysis of SO_2_, Hb, and flow vs. age. **(A)** Pre-surgery data. **(B)** All post-operative measurements pooled.

**FIGURE 7:**
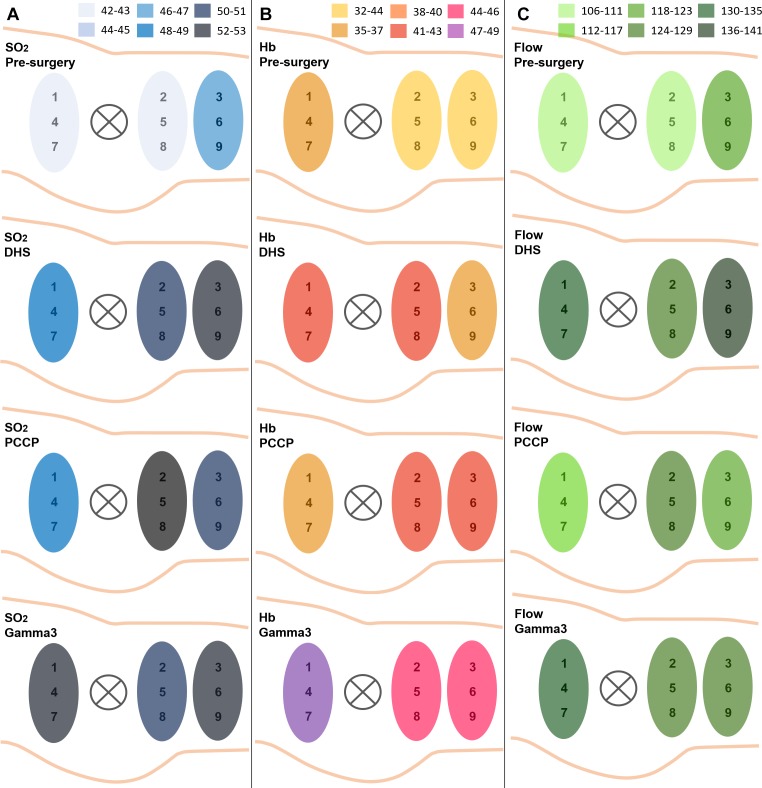
Average values of SO_2_ **(A)**, Hb **(B)**, and flow **(C)** for each implant (DHS, PCCP, and Gamma2 nail) displayed in three regions including three measurement points each. Average values are color-coded as shown.

Regarding Hb, its content was significantly influenced by depth (Figure 4B), age (Figure 6A), gender (Figure 3B), smoking (Figure 3E), and diabetes (Figure 3H) already before surgery. After surgery the categories implant (Figure 7), measurement point (Figure 7) and leg (Figure 2B) revealed significant influence as well, while the significant influence of diabetes disappeared (Figure 3H). Flow was significantly influenced by depth (Figure 4C) and diabetes (Figure 3I) already before surgery, whereas the factors measurement point (Figure 7), leg (Figure 2C), age (Figure 6B), and gender (Figure 3C, 5A) led to additional significant differences in flow after surgery.

### Time Course of Effects of Surgery

Figure 4 represents an overview of the values for SO_2_, Hb, and flow of the injured leg, averaged among the patient collective and separated by depth and time point. The SO_2_ values (Figure 4A) ranged between 20 and 70 AU, while in general, the 2 mm depth values were significantly lower than 8 and 15 mm values in the pre and in the post-surgery data (*p* < 0.001). The difference between the depth level after surgery partially disappeared when values of each depth almost merged 7 days after surgery (Figure 4A). In comparison to pre-surgery, a maximum drop in the 8 and 15 mm SO_2_ values by 26 and 39% on average, compared to pre-surgery, was observed, beginning 4 days after surgery. Such a drop was not present in the superficial skin layer (2 mm depth), where a rapid increase of 81% occurred during surgery, followed by a constant steady decline that did not return to pre-surgery values. SO_2_ values in all depths did not return to their baseline values 12 days after surgery.

Hb values (Figure 4B) ranged between 30 and 50 AU. Before surgery, 2 mm depth values were significantly higher compared to 8 and 15 mm values (*p* < 0.001). The former peaked 8 h after surgery with an increase by 18%. Difference in Hb between skin layers was existing only during the period starting before surgery and lasting until 24 h after surgery. Both 8 and 15 mm Hb values increased by 43% (Figure 2D) peaking 4 days after surgery.

Flow values differed significantly at each measurement depth before and after surgery (each *p* < 0.001, Table 2). Average values for each time point were around 20 AU in 2 mm, between 100 and 150 AU in 8 mm and between 200 and 310 AU in 15 mm depth. Compared to pre-surgery level, the greatest differences in 2 and 8 mm depth were observed 8 h after surgery and in 15 mm depth 48 h after surgery (Figure 4C). While little variation occurred over time in 2 mm depth, and in 8 mm depth, 15 mm showed a decrease in flow by 27% 48 h after surgery. The 15 mm blood flow was the only parameter to decrease after trauma while all other parameters increased (Figure 4).

### Effects of Gender

Findings revealed higher values in SO_2_ and flow in women compared to men before and after surgery. Figure 5A–D show SO_2_, Hb, and flow for all depths together (Figure 5A) and separated by measurement depth (2 mm Figure 5B, 8 mm Figure 5C and 15 mm Figure 5D). The more detailed analysis of each separate depth showed significantly higher SO_2_ values in women than men in all depths, while flow only differed significantly in 8 and 15 mm depth and showed no significant difference in 2 mm.

Data were further analyzed by separating the time points. Figure 3A–I present SO_2_, Hb, and flow values for all depths together over time, respectively. Figure 3C shows how differences is SO_2_ between genders remained stable over time. Higher SO_2_ values for women were consistent over all measurements in 8 mm (Supplementary Figure 7A) and 15 mm (Supplementary Figure 8A) depth, while not always in 2 mm depth (Supplementary Figure 6A). The analysis of Hb (Figure 3B) revealed higher Hb values in men up to 4 days after surgery and higher values in women 7 and 12 days after surgery. Analysis of flow (Figure 5A) revealed higher values for women on average. There were huge differences in flow in the first 48 h in 8 mm (Supplementary Figure 7C), while differences span all time points in 15 mm (Supplementary Figure 8C).

### Effects of Smoking

The analysis of all depths pooled together showed significantly higher flow values in smokers compared to non-smokers after surgery, but no differences in SO_2_ and Hb (Figure 5E). Analysis of data separated by depth, however, gave additional detail: in 2 mm, smokers had higher SO_2_, Hb, and flow values than non-smokers (Figure 5F). In 8 mm, SO_2_ values were significantly higher for non-smokers and flow values again significantly higher for smokers (Figure 5G). There were no effects of smoking in 15 mm depth (Figure 5H). Separating by time points suggested a delay or right-shift in SO_2_-response in smokers compared to non-smokers (Figure 3D). Comparison of separated depths, however, did not confirm this finding and explained it by an effect of summation (Supplementary Figures 6D, 7D, 8D).

### Effects of Diabetes

The analysis of all depths pooled (Figure 5I) showed significantly increased SO_2_ in diabetics compared to non-diabetics after surgery. Diabetes caused decreased flow in 2mm and 8mm depth and increased SO_2_ in 8 mm and 15 mm (Figure 5K–M).However, the difference in flow here was not significant (*p* = 0.078). The analysis of changes over time showed increased SO_2_ values in diabetes patients 24 h and 7 days after surgery that originated in 8 and 15 mm depth (Supplementary Figures 6G, 7G, 8G).

### Effects of Age

Figure 6 shows results from the regression analyses by plotting SO_2_, Hb, and flow against age. Figure 6A shows the pre-surgery data and Figure 6B all post-operative measurements pooled. While flow increased with age before surgery, it decreased with age after surgery. Before surgery, the regression line of flow showed a slope coefficient of 0.6511, and after surgery, the slope coefficient was −0.51. Of note, the R^2^ was very small (0.041 and 0.0939) as a sign of high variance. The absolute values were much higher after surgery as reflected in the y-intercept of the regression line: it was at 58.3 AU before and 163.5 AU after surgery. At age 95, the regression line was in the same area before and after surgery. These findings indicate an age-related increase in flow after surgery in the young patients that was not observed in the old patients. In other words, the ability to alter the flow rate after surgery decreased with age. In addition, the correlation of age with flow showed a significant difference (Table 2) after surgery and not before. Further regression analyses for each depth after surgery are presented in Supplementary Figure 1. Flow-decline was steepest in 8 mm depth (slope coefficient −0.7155).

### Comparison of Implants

Figure 7 shows a comparison of implants separating the nine measurement points by three regions and comparing pre-surgery and post-surgery values. All post-surgery regional averages (SO_2_, Hb, and flow) were increased compared to pre-surgery. There were no significant differences between areas pre-operatively (one-way ANOVA). Post-operative ANOVA-analyses of regions showed the following significant differences between implants: For SO_2_, the Gamma3 nail had significantly higher values than DHS (*p* = 0.042) and PCCP (*p* = 0.019) in the proximal region, while DHS had significantly higher values than PCCP in the distal region (*p* = 0.037). In Hb, the Gamma3 nail showed significantly higher values in the proximal region than the other two implants (both *p* < 0.001), and DHS had significantly higher values than PCCP (*p* = 0.007). Regarding flow, the only significant difference between regions found were higher values in DHS than PCCP in the distal region (*p* = 0.021).

In more depth, Supplementary Figure 2 shows a comparision of implants over time and Supplementary Figures 3–5 show average values of each implant for each measurement point and depth for SO_2_ (Supplementary Figure 3), Hb (Supplementary Figure 4), and flow (Supplementary Figure 5) and indicate significant differences between implants from one-way ANOVAs.

Apart from the finding of the measurement point analysis shown in the Supplementary Figures 3–5, a second one-way ANOVA analysis was conducted to compare the implants with all time and measurement points as well as depths pooled. Here, PCCP overall altered microcirculation the least. With respect to SO_2_ and Hb, average values over all postoperative time and measurement points did not differ between implants (average and SD: SO_2_: DHS 50.0 AU ± 25.2, PCCP 50.1 AU ± 24.9, Gamma3 nail 49.9 AU ± 25.1; Hb: DHS 40.5 AU ± 19.2, PCCP 39.5 AU ± 15.6, Gamma3 nail 42.8 AU ± 21.8). In flow, however, PCCP showed the lowest average values, which are the closest to pre-surgery values (Average and SD: Flow: DHS 134.1 AU ± 140.6, PCCP 120.6 AU ± 118.8, Gamma3 nail 131.1 AU ± 142.8, one-way ANOVAS: DHS/PCCP *p* = 0.001, PCCP/nail *p* = 0.005, DHS/nail *p* = 0.485).

## Discussion

The aim of the current study was to evaluate perioperative changes in microcirculation after trochanteric femur fractures by means of non-invasive laser-Doppler spectrophotometry. As no studies have previously investigated tissue microcirculation after fixation of proximal femur fractures or hip arthroplasty, the present data delivered totally new insights into pathophysiology and risk factors of such fractures. The following findings were obtained, and the null-hypothesis rejected.

(1) Surgery induced significant differences between the healthy and injured leg in SO_2_, Hb, and flow. Each parameter was with comparable values for both legs prior to surgery.
(2) Significantly higher values in SO_2_ and flow were registered in women compared to men before and after surgery.
(3) Smoking caused significant increases in SO_2_, Hb, and flow only in the superficial layer of the skin after surgery.
(4) Diabetes decreased blood flow at 2 and 8 mm depth and increased SO_2_ at 8 and 15 mm depth after surgery.
(5) Age revealed a significant negative correlation with flow. The ability to increase the flow rate after surgery decreased with age.
(6) Comparison of implants indicated the minimally invasive implant PCCP altered microcirculation less than the DHS or the Gamma3 nail.

### Interpretation of Measured Parameters

As the capillary system predominantly contains venous blood with desaturated hemoglobin after passing the tissue, the oxygen saturation there reflects the level of tissue hypoxia. Regional hemoglobin concentrations (Hb) correlate with postcapillary filling, which in its turn depends on the equilibrium of arterial influx and venous efflux and may reflect venous stasis or congestion. Blood flow describes the amount of blood passing in a certain period and can be interpreted as a global surrogate parameter for microvascular function (Forst et al., 2008). It depends on several factors including local temperature, systemic and local hormones, inflammation and inflammatory mediators and the sympathetic nervous system (Forst et al., 2008).

### No Effects of Injury Before Surgery

Before the surgery, microcirculation did not differ between the injured and healthy leg. This means, the proximal femur fracture alone did not alter local skin microcirculation significantly in a way comparable to the effect caused by surgery. This outcome is surprising, as cytokines and other inflammatory mediators are known to be released into the fracture hematoma and surrounding tissues (Horst et al., 2015). Thick local soft tissue layers of the hip might be an explanation for the finding, and changes in microcirculation might be measurable after fractures in body parts with less soft tissue cover, such as the lower leg or forearm. Apart from local changes, the systemic immune response mediated by cytokines following trauma would have affected both legs in the same way (Hildebrand et al., 2005).

Apart from the overall results, however, one finding indicated a possible effect of injury already before surgery: a pre-surgery elevation in 2 mm Hb. The fact that Hb 2 mm values decreased to 92% of pre-surgery values 12 days after surgery indicates a possible increase from physiological parameters already before surgery. This finding might reflect superficial skin vasodilation and is the only indication we found for differences between the healthy and injured leg before surgery.

### Effects of Surgery

Surgery caused changes in all measured microcirculatory parameters. To our knowledge, our study is the first to present details on changes in microcirculation after hip surgery. Differences were found in all parameters and in 2, 8, and 15 mm depth. These differences were particularly striking regarding blood flow. The 15 mm blood flow was the only parameter to decrease after trauma, while all other parameters in all depths increased. Reasons might be related to the anatomy of blood vessels. The capillaries of the skin are connected to arteries and veins with greater diameters in the subcutaneous layer. In the lateral hip, measurements in 15 mm depth represent the subcutaneous layer and display blood flow in the deep plexus. Here, muscle sphincters surround arterioles and venules, innervated by sympathetic nerves (Johnson et al., 2014). By these sphincters, blood flow may be controlled and re-directed. In our case of decreasing flow values in 15 mm, blood flow seems to have been bypassed from the deep plexus into the capillaries of the more superficial skin layers. Blood flow in 2 mm increased.

Apart from changes in blood flow, another effect we observed was an increase in SO_2_ values in the injured leg during the first 48 h, followed by a sudden drop in SO_2_ values most prominent 7 days after surgery. The increase was mainly caused in the 2 mm layer, while the drop was caused in the 15 mm layer. Superficial skin SO_2_ values were probably increased by higher blood flow initially after surgery. The delayed drop in SO_2_ in the 15 mm layer, however, requires a separate explanation. The question is why the drop is delayed and does not appear immediately after surgery. A drop in SO_2_ reflects a local increase in hypoxia. A possible explanation for the finding is the course of the inflammatory process that requires more oxygen several days after surgery when inflammation reaches its most active state. Wound healing is the interplay of a multitude of cells and mediators (Sorg et al., 2017). Between days 3 and 10, during the proliferative phase, increasing numbers of cells involved in the healing process require growing amounts of oxygen.

### Gender

Higher rates of periprosthetic joint infection in male compared to female patients are known following total hip and knee arthroplasty (Kong et al., 2017). To the best of our knowledge, gender differences in microcirculation in relation to hip surgery have not been previously reported. In a study on microcirculation in the hindfoot with healthy subjects, higher values were registered for men than women (Carow et al., 2018). Reasons might include more subcutaneous fat tissue in women compared to men (Carow et al., 2018). Our finding of significantly increased values in SO_2_ and flow in women compared to men after proximal femur fractures is new, and further investigations are necessary to confirm these results. In case of confirmation of gender differences in microcirculation, data would be relevant for more personalized treatment and intervention pathways. Possible reasons for gender differences in this regard may be of hormonal nature and possibly related to estrogen and androgen concentrations (Panazzolo et al., 2013; Angele et al., 2014). Differences in smoking habits between genders are unlikely to be considered as a reason, as changes in microcirculation due to smoking differed from those related to gender. Flow differences between smokers and non-smokers were found in the superficial skin layer (2 mm depth), whereas differences between genders were registered in the deeper tissue layers (8 and 15 mm depth). In addition, gender differences regarding SO_2_ were found in all depths while smoking caused changes only in the superficial skin (2 mm depth). The same statement is valid for the other risk factors (such as diabetes and age). Therefore, they show different patterns of changes in comparison to gender and cannot serve as an explanation either.

### Smoking

Smoking decreases tissue oxygenation and quitting smoking reduces postoperative complications by 41% (Jensen et al., 1991; Mills et al., 2011). Nicotine causes vasoconstriction by increased levels of circulating epinephrine, norepinephrine and vasopressin, and thereby decreases microcirculation (Cryer et al., 1976; Waeber et al., 1984; Netscher et al., 1995). In addition, carbon monoxide is created when burning a cigarette and binds with hemoglobin, forming carboxyhemoglobin (COHb) and methemoglobin (MetHb), which reduces oxygen transport capacity (Krupski, 1991; Gavrilovska-Brzanov et al., 2017). Tabacco smoke contains up to 5% carbon monoxide (Goldsmith and Landaw, 1968) and smokers frequently have blood COHb concentrations of 5.5% (Hart et al., 2006). Higher tissue hemoglobin levels in smokers may therefore wrongly indicate a higher oxygen transport capacity than the actual one. Reductions of blood flow in the hands have been demonstrated in smokers during and within 20 min. after smoking e-cigarettes in an O2C-study (Pywell et al., 2018). Changes were more pronounced in superficial (77% reduction) compared to deep (29% reduction) blood flow. In the same study, smoking an e-cigarette without nicotine resulted in a flow increase of up to 50% in the superficial skin layer. Monfrecola et al. (1998) found a faster recovery of microcirculation in non-smokers than smokers after smoking a cigarette. Our study reports higher flow values in smokers compared to non-smoker in the superficial skin layer. This is in line with the findings of Pywell et al. (2018), as the smokers in our study had not smoked for a longer period before the measurement, presumably longer than 20 min. In addition, both studies found the strongest effects of smoking on blood flow in the superficial skin layer.

### Diabetes

Diabetes is one of the major risk factors for periprosthetic infections in hip and knee arthroplasty (Kong et al., 2017). Peripheral vascular and especially endothelial cell dysfunction in diabetes mellitus leads to micro- and macrovascular complications (Forst et al., 2008). Damage to the endothelium is probably mediated by glucose-level related damages to the gycocalyx (Lemkes et al., 2012; Zhang et al., 2018). Out results indicate significantly decreased flow and increased SO2 in diabetic patients. In an O2C-study by Forst et al. (2008), no significant differences in microcirculation were found between the hands of diabetics and non-diabetics. Measurements were, however, obtained from the hands of healthy (non-fractured) subjects, while we measured in patients with a proximal femur fracture on the skin close to this fracture site.

### Age

Microcirculation is known to deteriorate with age (Li et al., 2006), decreasing by 40% between 20 and 70 years (Tsuchida, 1993). The pathomechanism is probably linked to a diminished endothelial glycocalyx, just like in diabetes (Machin et al., 2018). Age is a risk factor for wound infections after primary total hip and knee arthroplasty (Kong et al., 2017). From an outcomes-based perspective, the health-related quality of life after femoral neck fractures was shown to decrease with age (Sprague et al., 2018). The present study revealed a significant decrease in blood flow with age after surgery, which is, despite a high variance, in line with the reports in the literature. The values did not differ from results in other body parts such as the hindfoot, where flow decreased with age in the 2 mm skin layer in all studies (Park et al., 1997; Forstmeier et al., 2015; Carow et al., 2018). Despite these matching results, however, we found that flow increased with age before surgery and that the ability to alter the flow rate after surgery decreased with age. This is a surprising result, as it has, to our knowledge, not been previously reported in connection to microcirculation. When considering diminished endothelial wall properties at higher age, a possible pathomechanism might be that the ability to regulate blood vessel diameters to in- or decrease microcirculation vanishes at higher age, and thereby the ability to react in acute situations. Some studies also reported lower skin oxygen saturation levels in older compared to younger patients (Ogrin et al., 2005; Bentov and Reed, 2014). Interestingly, SO_2_ and Hb were not affected by age in our study.

### Implants

In the present study, three implants were compared in nine measurement points surrounding the greater trochanter: the extramedullary implants DHS and PCCP (minimally invasive) and the intramedullary Gamma3 nail. Each implant requires different skin incisions and surgical approaches varying in locations, extent and lengths (Figure 1). Comparison of implants indicated the minimally invasive implant PCCP overall altered microcirculation the least. This conclusion is mainly based on its reduced impairment of blood flow. The finding is in line with the concept of shorter skin incisions and a tunneling of soft tissues (Knobe et al., 2012). The PCCP was developed to decrease surgical trauma, operation time and overall invasiveness especially with regards to geriatric patients for whom high mortality rates were reported in proximal hip fractures (Knobe and Siebert, 2014; Neuerburg et al., 2015). The authors, however, concluded no major differences between the implants in terms of wound infection, mortality or medical complications. Recent studies evaluating the minimally invasive PCCP, however, indicated lower complication rates compared to the other implants (Knobe et al., 2009, 2012, 2015). The present findings on microcirculation support this observation.

Overall, the findings in our study should be adopted in the development of new implants. Following from our results, minimally invasive techniques such as the PCCP ought to be further developed in a way that reduces the length and number of skin incisions even further to decrease disturbances in microcirculation to an even higher extent.

### Possible Perioperative Interventions

To mitigate reductions in microcirculation and decrease the risk of complications after treatment of proximal femur fractures, countermeasures known to improve the local vascular situation may be applied, especially in patients with a combination of several risk factors. Interventions include normothermia, oxygen supply, restrictions in fluid intake, pain control and nicotine cessation as well as avoidance of reductions in cardiac output and arterial pressure that directly affect skin blood flow (Bentov and Reed, 2014). Tissue oxygenation is greater at higher temperatures (Kakihana et al., 1998). Some medications including inhalation anesthetics such as halothane, enflurane and isoflurane are known to dose-dependently induce peripheral vasodilation and thereby improve microcirculation (Longnecker, 1984).

### Limitations

There were several dropouts related to the advanced measurement time points that reduced data availability especially for analysis of the effects 7 and 12 days after surgery. Another limitation is the short follow-up time of maximum 12 days that was not sufficient to show full recovery with regards to the measured parameters. Results indicate much longer recovery times for microcirculation after surgery of proximal femur fractures, probably several weeks or even months. A longer follow-up period should be considered in future studies. Another shortcoming is the low number of smokers and diabetics in the patient collective. In addition, laser-Doppler spectrophotometry is known to show substantial spatial, temporal, and intra-individual variation, which is a known weakness of this method (Sommer et al., 2013; Kösters et al., 2017).

## Conclusion

In conclusion, microcirculation after proximal femur fractures is highly altered by surgery, gender, smoking, diabetes and age. As no studies have previously investigated tissue microcirculation after fixation of proximal femur fractures, the present data delivered new insights into pathophysiology and risk factors. The comparison of implants and differences between measurement points indicate the least alteration of microcirculation by the minimally invasive implant PCCP, compared to DHS and the Gamma3 nail. The findings of the study support further reductions in invasiveness in terms of length and number of skin incisions to decrease the proven disturbances in microcirculation as much as possible. Decreasing superficial flow values with age, gender differences in SO_2_ concentrations and blood flow, as well as smoking-related significant increases in SO_2_, Hb, and flow in the superficial skin-layer were in line with previous results. The finding that the ability to increase the flow rate after surgery decreased with age and the findings on microcirculation in diabetic patients were previously not reported for surgical wounds. Decreased blood flow in the skin and increased SO_2_ in deeper tissue layers were new findings in connection to diabetes. In addition, the study identified groups particularly at risk for microcirculatory impairments, which is relevant with regards to interventions and personalized treatment pathways. O2C-measurements might be suitable for implementation in a clinical setting to monitor wounds and identify problems early enough to start an intervention. Interventions may include calculated antibiotic therapy or hyperbaric oxygen therapy. Further studies therefore need to validate the predictive value of O2C measurements for infections and wound healing delays/disorders in a clinical setting. In addition, effects of medications such as catecholamines on local microcirculation could be studied or maybe even monitored during treatment.

## Author Contributions

BGa contributed data analysis and interpretation, figures, tables, drafting, and approval of manuscript, manuscript submission. FB and TP contributed data collection and approval of the manuscript. BGu contributed data analysis, drafting, and approval of the manuscript. SA contributed drafting and spproval of the manuscript. GG contributed study conception, data collection, and approval of the manuscript. B-SK, AM, and SN contributed data interpretation, drafting, and approval of the manuscript. FH contributed data interpretation, organizational support, and approval of the manuscript. MK contributed the idea, organization, interpretation, drafting, and final approval of the manuscript.

## Conflict of Interest Statement

The authors declare that the research was conducted in the absence of any commercial or financial relationships that could be construed as a potential conflict of interest.
