# Supplementary material for: Microcirculation After Trochanteric Femur Fractures: A Prospective Cohort Study Using Non-invasive Laser-Doppler Spectrophotometry
Source: Front Physiol. 2019 Mar 25;10:236. doi: 10.3389/fphys.2019.00236 (PMC6442516; doi:10.3389/fphys.2019.00236)
Supplement: Supplementary file 1 [file Table_1.DOCX]

Supplementary information

**Microcirculation after proximal femur fractures: a prospective cohort study using non-invasive laser-Doppler spectrophotometry**

Ganse B et al.


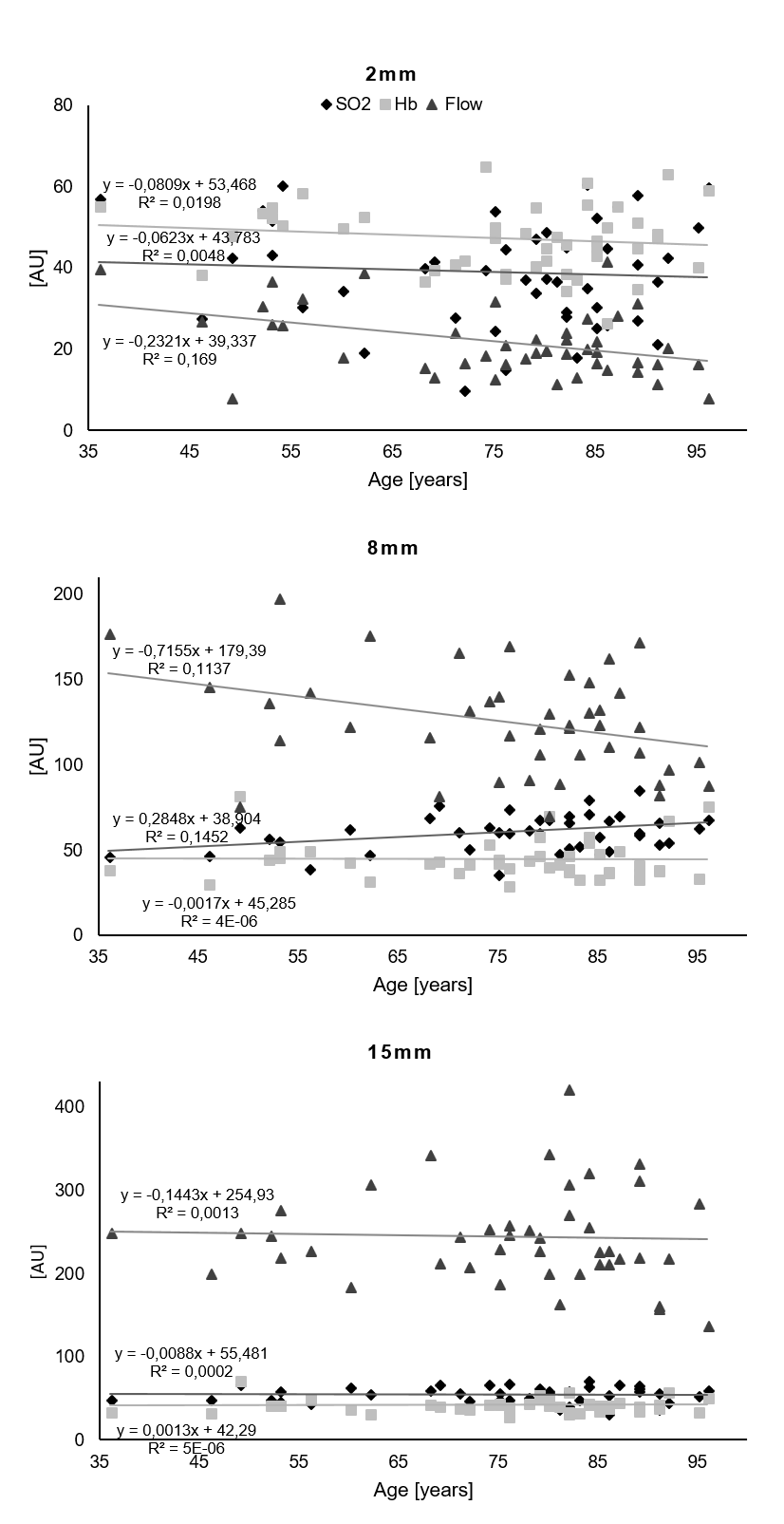


**Supplement figure 1:** Regression analysis of SO_2_, Hb and flow vs age for each depth.


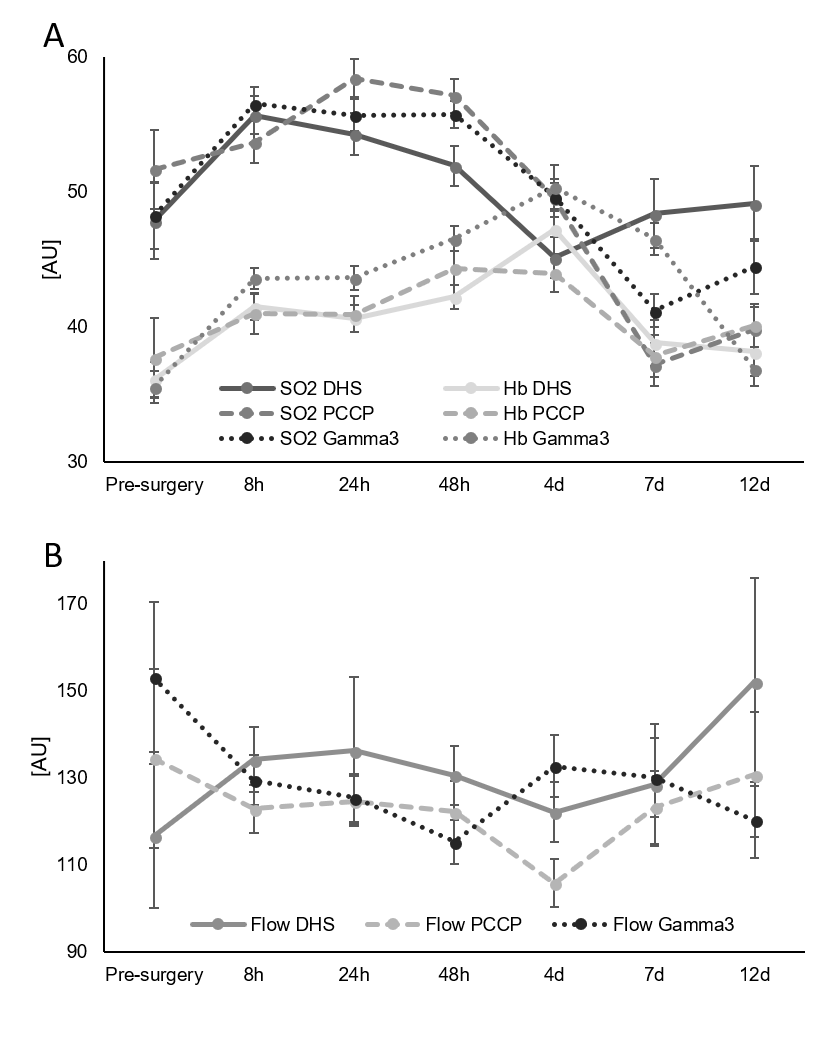


**Supplement Figure 2:** Comparison of implants over time. Error bars show SE. **A**: SO_2_ and Hb. **B**: flow.


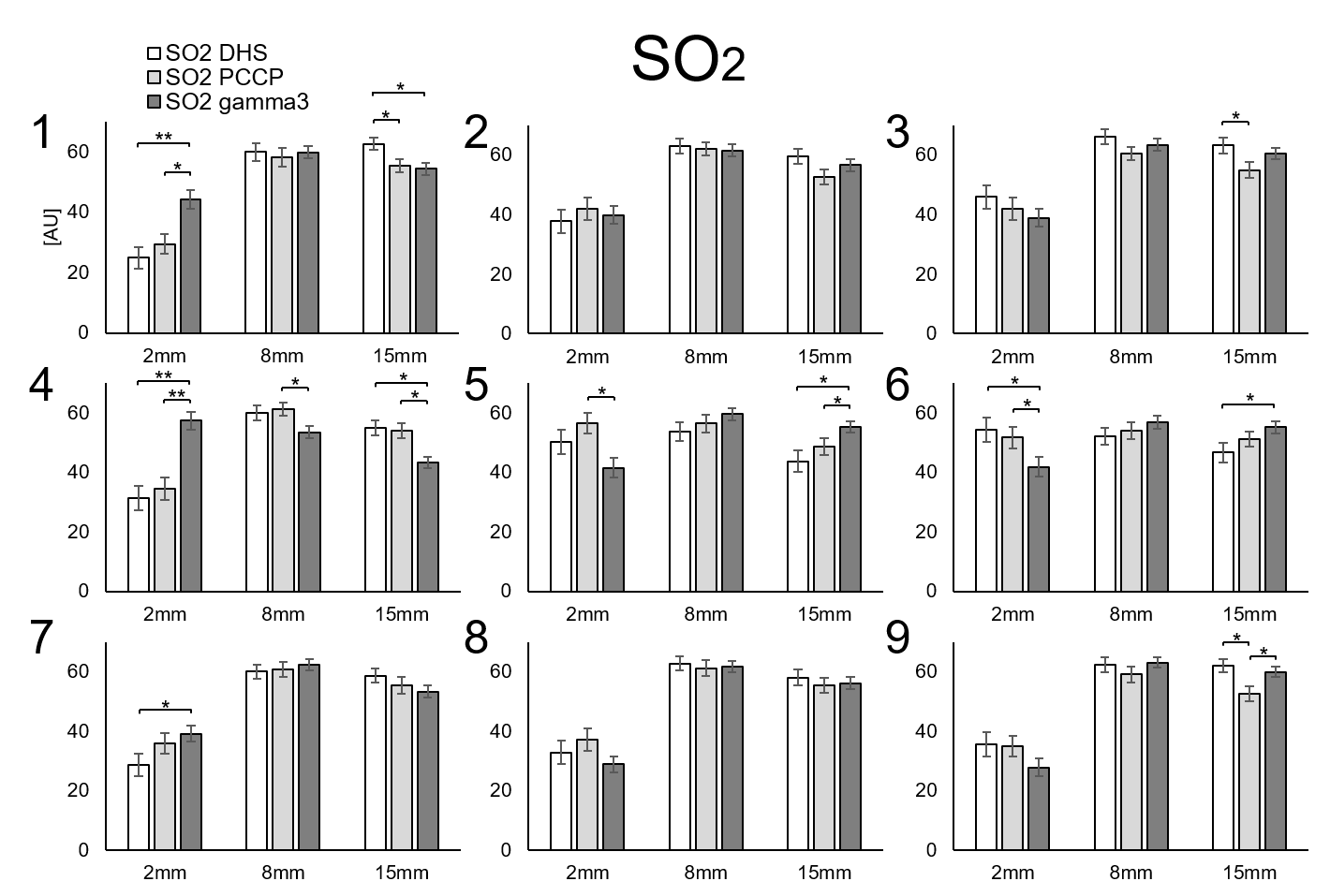
**Supplement figure 3:** Detailed analysis of SO_2_ for each implant (DHS, PCCP and Gamma2 nail) separated by measurement point 1-9 and depth. Error bars show SEM. * p < .05; ** p < .001 (one-way ANOVA)


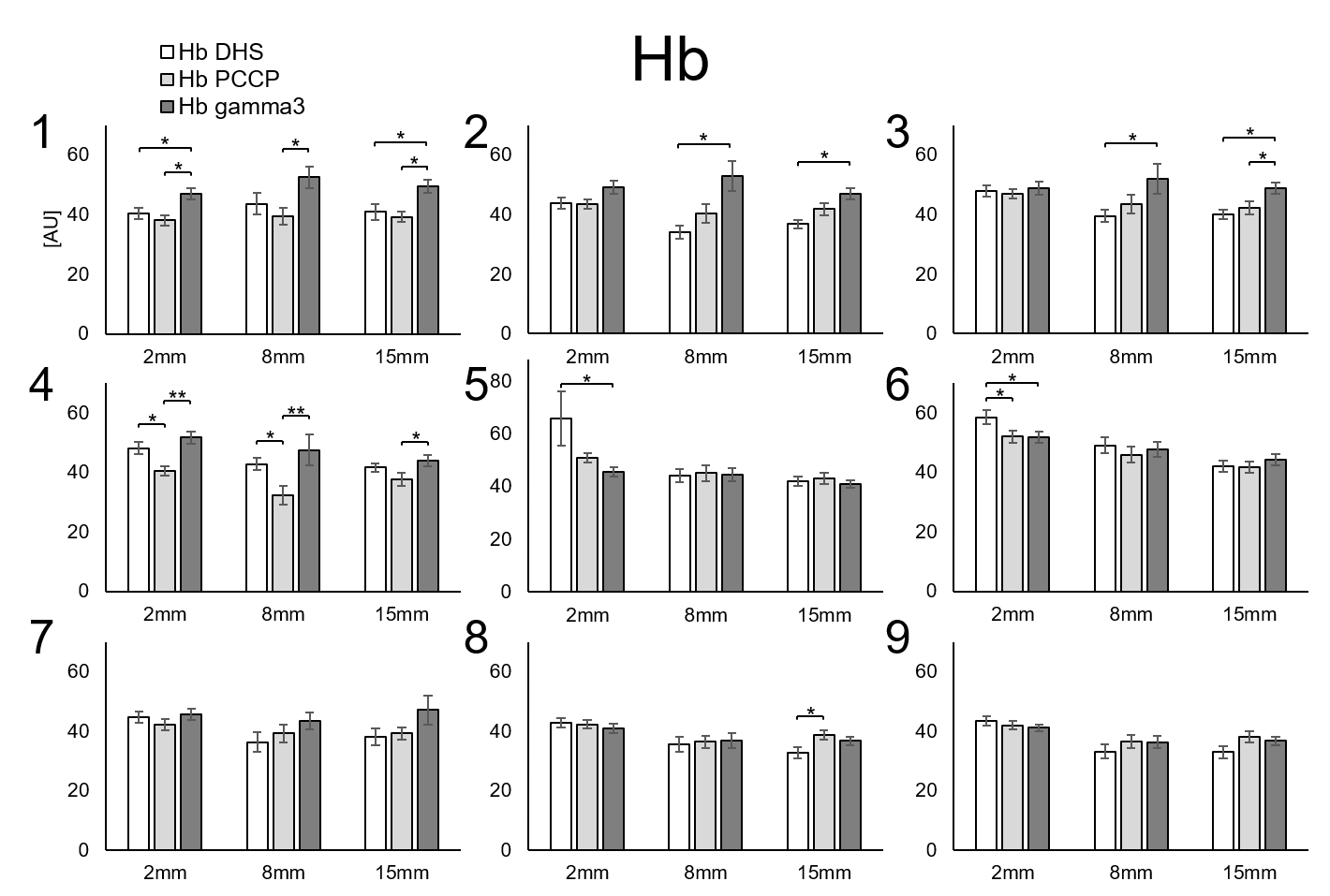
**Supplement figure 4:** Detailed analysis of Hb for each implant (DHS, PCCP and Gamma2 nail) separated by measurement point 1-9 and depth. Error bars show SEM. * p < .05; ** p < .001 (one-way ANOVA)


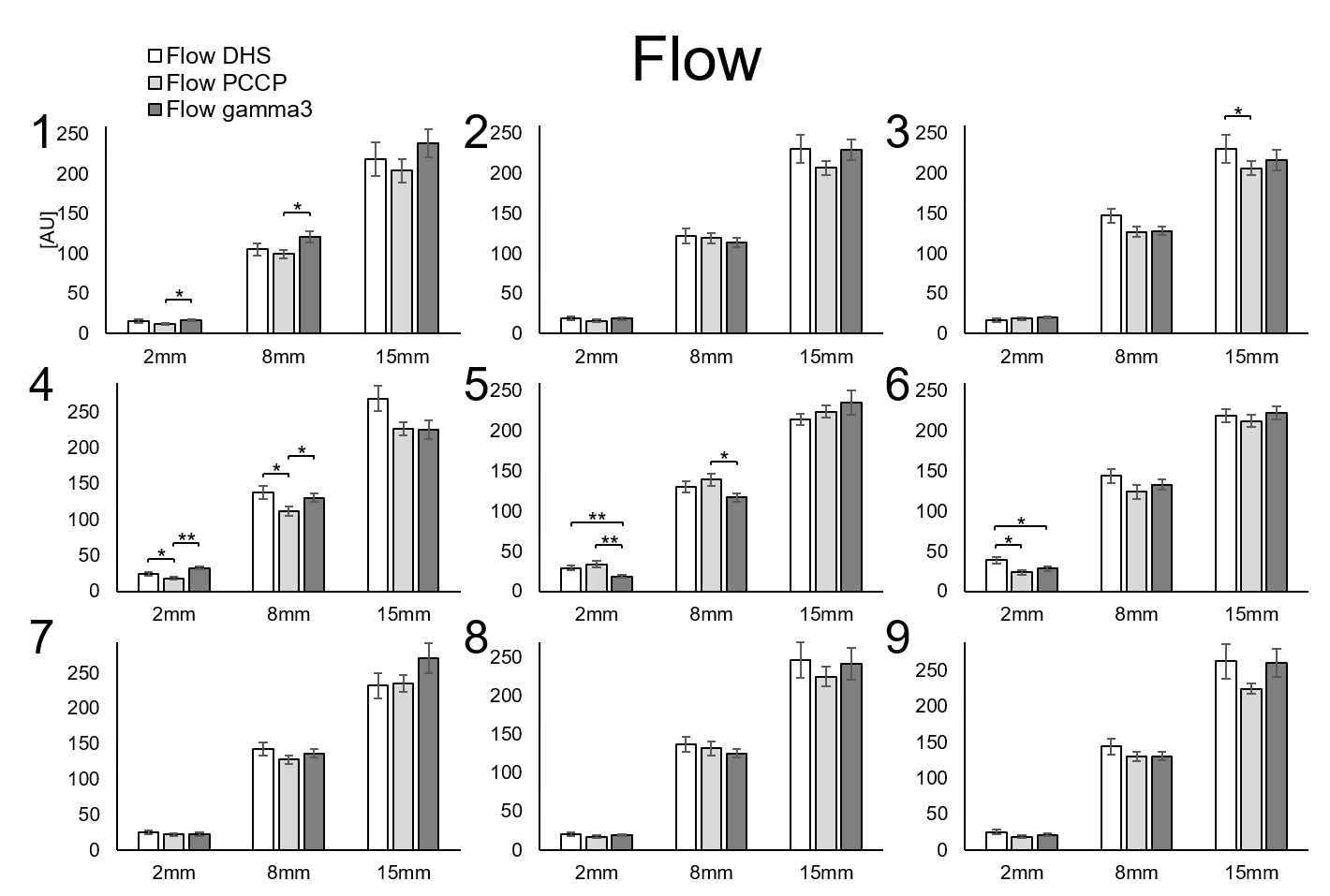
**Supplement figure 5:** Detailed analysis of flow for each implant (DHS, PCCP and Gamma2 nail) separated by measurement point 1-9 and depth. Error bars show SEM. * p < .05; ** p < .001 (one-way ANOVA)


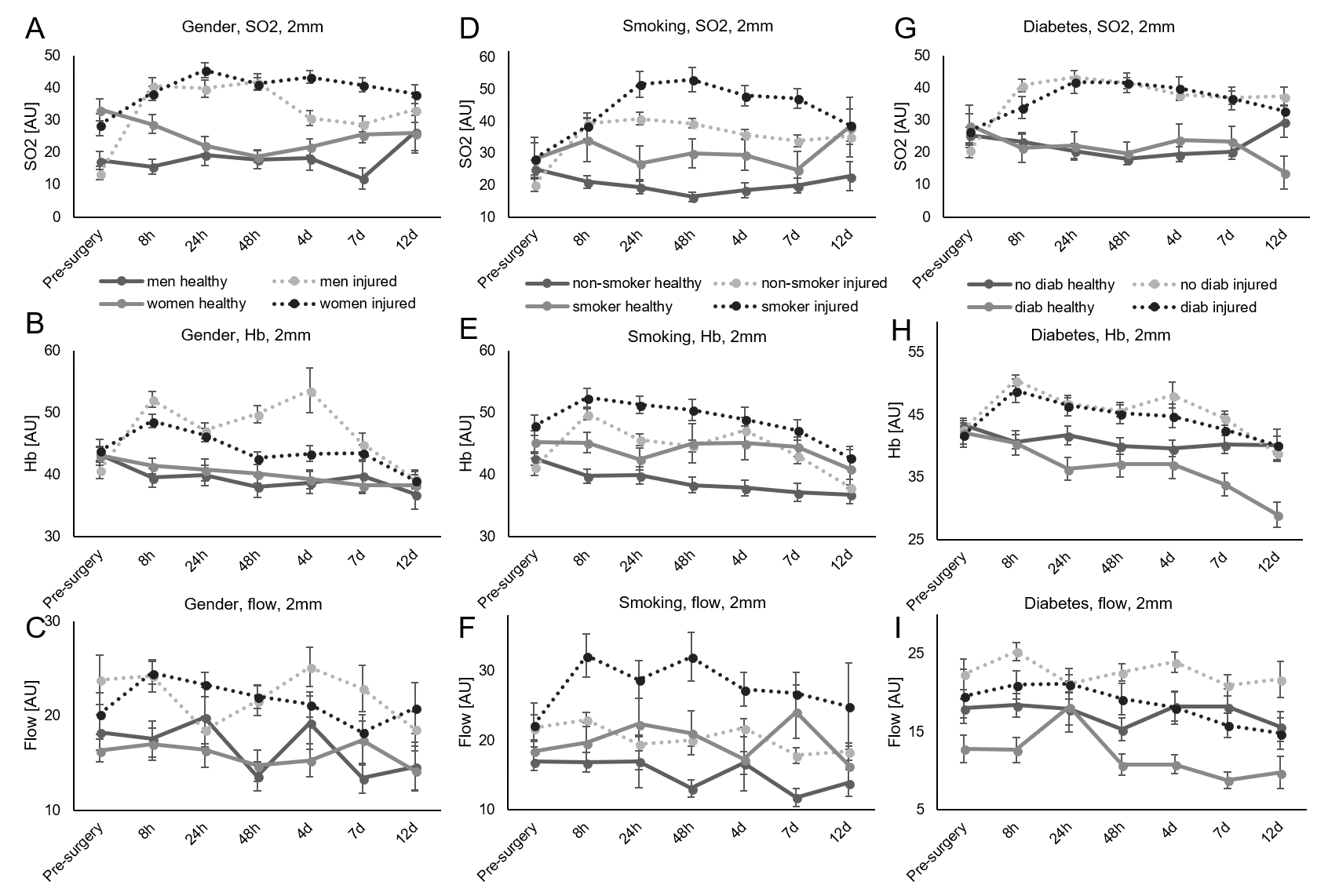


**Supplement figure 6:** Comparison of healthy and injured leg shown for each measurement time point for 2 mm depth. Error bars show SEM. **A-C**: Gender, **D-F**: Smoking, **G-I**: Diabetes.


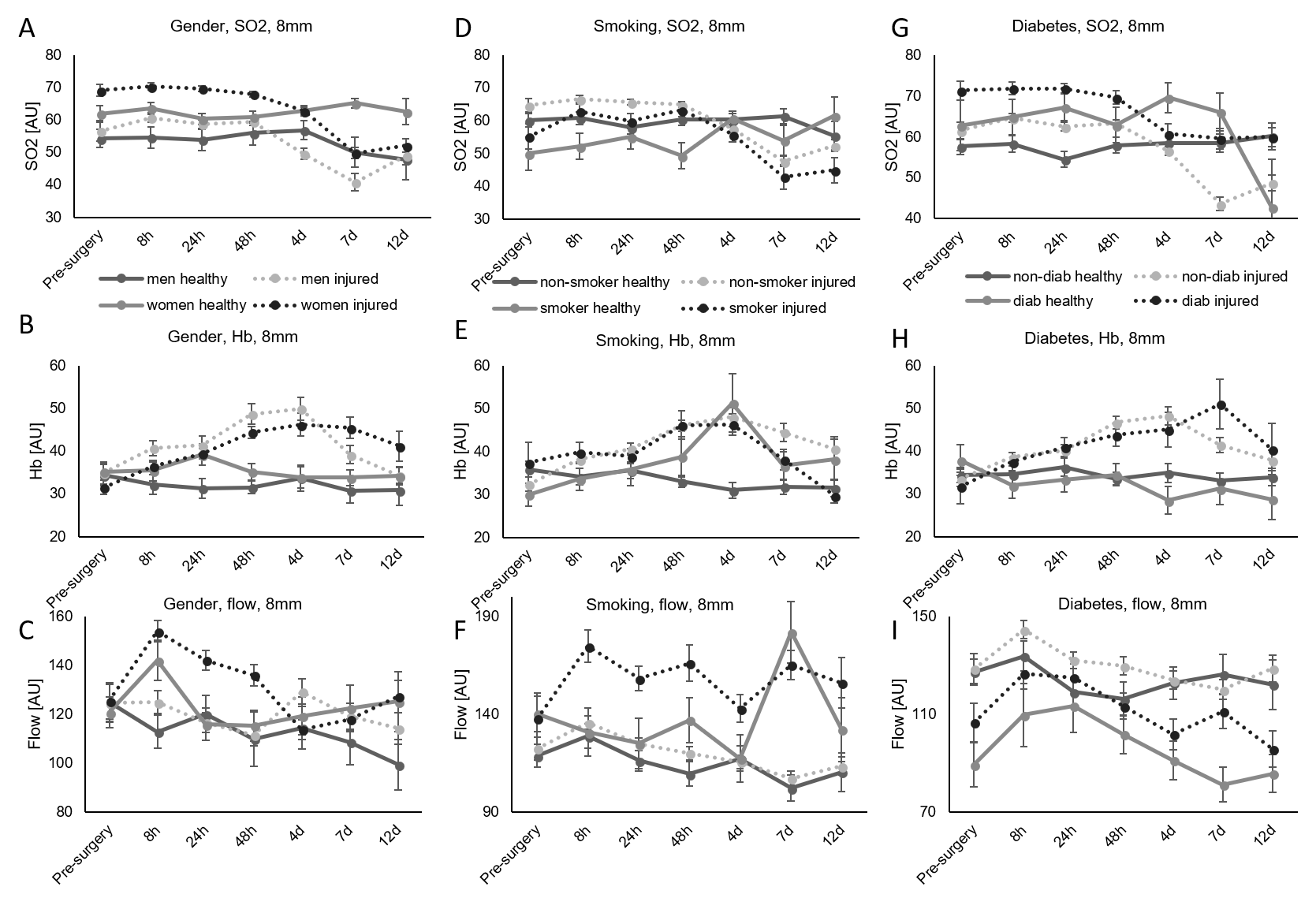


**Supplement figure 7:** Comparison of healthy and injured leg shown for each measurement time point for 8 mm depth. Error bars show SEM. **A-C**: Gender, **D-F**: Smoking, **G-I**: Diabetes.


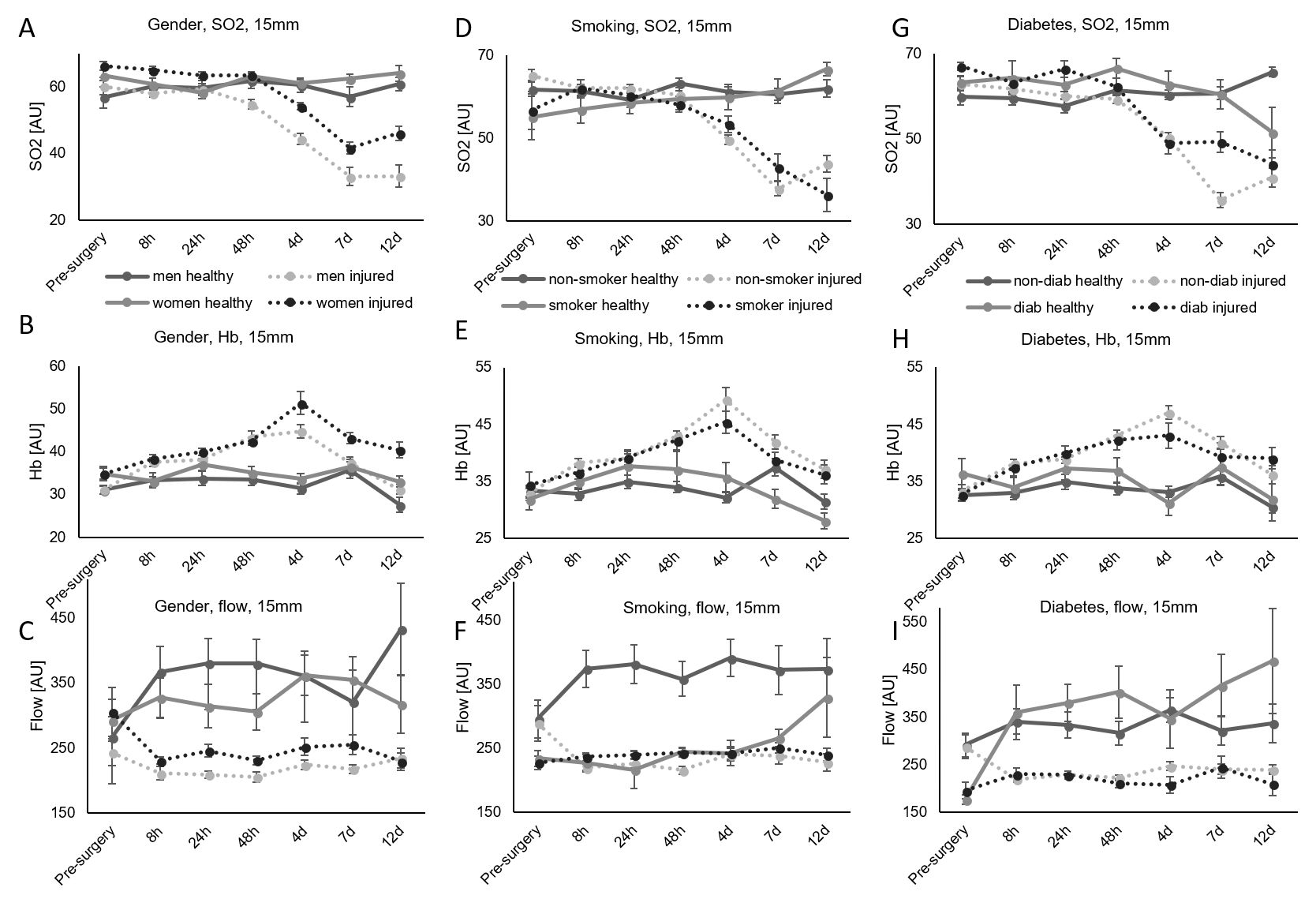


**Supplement figure 8:** Comparison of healthy and injured leg shown for each measurement time point for 15 mm depth. Error bars show SEM. **A-C**: Gender, **D-F**: Smoking, **G-I**: Diabetes.
